# Pair Distribution Function Analysis of ZrO_2_ Nanocrystals and Insights in the Formation of ZrO_2_-YBa_2_Cu_3_O_7_ Nanocomposites

**DOI:** 10.3390/ma11071066

**Published:** 2018-06-23

**Authors:** Hannes Rijckaert, Jonathan De Roo, Matthias Van Zele, Soham Banerjee, Hannu Huhtinen, Petriina Paturi, Jan Bennewitz, Simon J. L. Billinge, Michael Bäcker, Klaartje De Buysser, Isabel Van Driessche

**Affiliations:** 1 Sol-gel Centre for Research on Inorganic Powders and Thin films Synthesis (SCRiPTS), Department of Chemistry, Ghent University, Krijgslaan 281-S3, 9000 Ghent, Belgium; Hannes.Rijckaert@ugent.be (H.R.); Jonathan.DeRoo@ugent.be (J.D.R.); Matthias.VanZele@ugent.be (M.V.Z.); Klaartje.DeBuysser@ugent.be (K.D.B.); 2 Department of Applied Physics and Applied Mathematics, Columbia University, 1105 S.W. Mudd, New York, NY 10027, USA; sb3519@columbia.edu (S.B.); sb2896@columbia.edu (S.J.L.B.); 3 Wihuri Physical Laboratory, Department of Physics and Astronomy, University of Turku, 20014 Turku, Finland; Hannu.Huhtinen@utu.fi (H.H.); Petriina.Paturi@utu.fi (P.P.); 4 BASF SE, Advanced Materials & Systems Research, Carl-Bosch-Straße 38, 67056 Ludwigshafen am Rhein, Germany; Jan.Bennewitz@basf.com; 5 Condensed Matter Physics and Materials Science Department, Brookhaven National Laboratory, Upton, NY 11973, USA; 6 Deutsche Nanoschicht GmbH, Heisenbergstraβe 16, 53359 Rheinbach, Germany; Baecker@d-nano.com

**Keywords:** chemical solution deposition, nucleation and growth, nanocomposite, thin film, YBa_2_Cu_3_O_7−δ_, superconductor, nanoparticles, SIMS

## Abstract

The formation of superconducting nanocomposites from preformed nanocrystals is still not well understood. Here, we examine the case of ZrO_2_ nanocrystals in a YBa_2_Cu_3_O_7−x_ matrix. First we analyzed the preformed ZrO_2_ nanocrystals via atomic pair distribution function analysis and found that the nanocrystals have a distorted tetragonal crystal structure. Second, we investigated the influence of various surface ligands attached to the ZrO_2_ nanocrystals on the distribution of metal ions in the pyrolyzed matrix via secondary ion mass spectroscopy technique. The choice of stabilizing ligand is crucial in order to obtain good superconducting nanocomposite films with vortex pinning. Short, carboxylate based ligands lead to poor superconducting properties due to the inhomogeneity of metal content in the pyrolyzed matrix. Counter-intuitively, a phosphonate ligand with long chains does not disturb the growth of YBa_2_Cu_3_O_7−x_. Even more surprisingly, bisphosphonate polymeric ligands provide good colloidal stability in solution but do not prevent coagulation in the final film, resulting in poor pinning. These results thus shed light on the various stages of the superconducting nanocomposite formation.

## 1. Introduction

Generators and other rotating devices used in energy conversion are important applications for the high-temperature YBa_2_Cu_3_O_7−δ_ (YBCO) superconductor [1,2]. However, these devices operate under magnetic fields that reduce the performance of the superconductor significantly due to vortex motion [2]. The incorporation of nano-sized structural defects as so-called ‘artificial pinning centers’ in the YBCO matrix can immobilize the vortices and thus create effective ‘pinning’ [3]. YBCO nanocomposite films, grown via pulsed laser deposition, were shown to maintain high critical currents in high magnetic fields. In these nanocomposite films, non-superconducting BaMO_3_ nanocolumns (M = Zr, Hf and Sn) are created in the YBCO matrix by manipulating the film deposition process [4,5]. Nanocolumns generate a good in-field performance when the magnetic field (*H*) is aligned parallel to them (i.e., *H*||*c*-axis). Such defects are correlated pinning centers along the *c*-axis and produce an enhancement of critical current densities (*J*_c_) at *H*||*c* with a pinning force density of more than 25 GN m^−3^ at 77 K [5]. To increase *J*_c_ at all orientations with respect to the magnetic field, non-correlated pinning centers (Y_2_O_3_ or BaCeO_3_) were introduced as nanodots [6,7]. Pulsed laser deposition-based coatings thus already feature an intricate control over the size, shape, and density of pinning centers [4,8,9,10].

However, chemical solution deposition of YBCO films with embedded nanoparticles offers higher deposition speeds and lower processing costs. At first, nanocomposites were synthesized by a spontaneous segregation of secondary phases in the YBCO matrix, due to excess metal salts in the precursor. In this way, a variety of secondary phases were grown: Y_2_O_3_, BaZrO_3,_ BaHfO_3_, BaCeO_3_, and Ba_2_YTaO_6_) [11,12,13,14,15,16]. This approach enhances in-field performances compared to undoped YBCO films but the pinning force densities (77 K, *H*||*c*) are still lower than the nanocomposite films by pulsed laser deposition. To improve performance, the nanoparticles must remain small (3–10 nm) and homogenously distributed in the YBCO matrix. In this respect, spontaneous segregation offers limited control on the formation and size distribution of the nanostructures and faces issues with reproducibility. To reproducibly gain control over the final microstructure of the nanocomposite films, we have pioneered the introduction of colloidally stable nanocrystals in the YBCO precursor solution [17,18]. A key advantage of this approach is the improved control over the composition, the particle size, and the concentration of the nanocrystals [19]. Using preformed nanocrystals, both trifluoroacetic-based and low-fluorine YBCO nanocomposite films were produced and showed a successful increase of the pinning force [17,18,19].

It was demonstrated that the nanocrystal surface chemistry is a crucial parameter influencing the nanocomposite formation. We determined via X-ray diffraction (XRD) and thermogravimetrical studies that the choice of stabilizing ligands affects the YBCO growth. However, it is not entirely clear how the various ligands affect the growth.

In this work, we first extensively characterize the ZrO_2_ nanocrystals, synthesized in trioctylphosphine oxide. Although their native surface chemistry was already elucidated, their crystal structure was ambiguous. Here, we firmly established the nanocrystals as tetragonal and exceptionally well ordered, comparable to bulk single crystals. Second, we exchange the native hydrophobic ligands for various polar surface ligands to provide dispersibility in the methanol-based YBCO precursor solution. Subsequently, we use secondary ion mass spectroscopy (SIMS) to determine the impact of these various ligands on the distribution of metal ions in the YBCO matrix after deposition and pyrolysis of the precursor. We find that the homogeneous distribution of Ba/Y in the pyrolyzed matrix is an important requirement as it has an influence on the YBCO growth and thus also on the final superconducting properties. Moreover, the use of a phosphonate-containing copolymer ligand leads to an epitaxial YBCO structure with good superconducting properties in spite of the presence of phosphorus and thus possible degradation of superconducting properties. Even though a copolymer with a bisphosphonate group can be used as ligand and does not affect the YBCO growth, the final YBCO layer exhibits no improvement of pinning behavior as a function of the magnetic field. This is likely due to the loss of ligand stabilization from ZrO_2_ nanocrystals during the pyrolysis step, leading to large coagulated BaZrO_3_ particles in the size range of 150–200 nm. This comprehensive study provides strategies towards improving the superconducting properties of YBCO nanocomposite films and controlling pinning behavior by the careful choice of ligands.

## 2. Materials and Methods

### 2.1. Nanocrystal Synthesis and Stabilization

ZrO_2_ nanocrystals in toluene were synthesized and purified according to De Keukeleere et al. [20]. In a ligand exchange step to polar solvents, 1 mL (0.3 mmol) ZrO_2_ dispersion is first precipitated by addition of acetone (1:3 by volume). In a second step, the precipitate (obtained after centrifugation at 5000 rpm for 2 min) is transferred to 1 mL methanol via the addition of a 35 mg phosphonate-containing copolymer or 15 mg short carboxylate, leading to a transparent and stable nano-suspension after an ultrasonic treatment of 30–60 min, according to previous work [17].

### 2.2. Nanocrystal Characterization

The solvodynamic diameter of the nanocrystals was determined via Dynamic light scattering (DLS) analysis on a Malvern Nano ZS (Malvern, United Kingdom) in backscattering mode (173°). Nuclear Magnetic Resonance (NMR) measurements were recorded on a Bruker Avance II Spectrometer (Billerica, MA, USA) operating at a ^1^H and ^13^C frequency of 500.13 and 125.77 MHz respectively and featuring a ^1^H, ^13^C, ^31^P TXI-Z probe. The sample temperature was set to 298.15 K. Diffusion measurements (2D DOSY) were performed using a double stimulated echo sequence for convection compensation and with monopolar gradient pulses. Smoothed rectangle gradient pulse shapes were used throughout. The gradient strength was varied linearly from 2–95% of the probe’s maximum value (calibrated at 50.2 G cm^−1^) in 64 steps, with the gradient pulse duration and diffusion delay optimized to ensure a final attenuation of the signal in the final increment of less than 10% relative to the first increment. The diffusion coefficients were obtained by fitting the appropriate Stejskal-Tanner equation to the signal intensity decay. Total scattering x-ray measurements were performed at the National Synchrotron Light Source II (XPD, 28-ID-2), Brookhaven National Laboratory (Upton, NY, US). Nanocrystalline powders of ZrO_2_ were sealed in polyimide capillaries and diffraction patterns were collected at room temperature in a transmission geometry with an X-ray energy of 66.47 keV (λ = 0.1866 Å) using a large-area 2D PerkinElmer detector (Waltham, MA, USA). The detector was mounted with a sample-to-detector distance of 202.99 mm. The experimental geometry, 2θ range, and detector misorientations were calibrated by measuring a crystalline nickel powder directly prior to the zirconia nanocrystals, with the experimental geometry parameters refined using the Fit2D program [21]. Standardized corrections are then made to the data to obtain the total scattering structure function, *F*(*Q*), which is then Fourier transformed to obtain the Pair Distribution Function (PDF), using PDFgetX3 [22] within xPDFsuite [23] The maximum range of data used in the Fourier transform (*Q*_max_, where *Q* = 4 πsinθ/λ is the magnitude of the momentum transfer on scattering) was chosen to be 23.5 Å to give the best tradeoff between statistical noise and real-space resolution. The PDFgui program was used to construct virtual crystal (VC) nanoparticle models from reference structures, carry out refinements, and determine the agreement between calculated PDFs and data, quantified by the residual [24]. Starting structure models for three bulk crystallographic phases of ZrO_2_ were obtained from single crystal and neutron diffraction studies established in the literature [25,26]. For the tetragonal model, refined atomic coordinates are reset to their high symmetry positions prior to the first refinement (P4_2_/nmc-I, Table 1). Refinements of the candidate phases are kept conservative. Lattice parameters and bond angles are constrained by symmetry, one isotropic atomic displacement parameters (ADPs) is applied per element (Zr, O), and a spherical particle diameter (SPD) is refined to account for the finite size of the nanocrystals [27]. Atomic positions are not refined unless specified and occupancies are kept full.

### 2.3. Thin Film Deposition and Processing

YBCO precursor solution is prepared by dissolving barium trifluoracetic, copper propionate, and yttrium propionate in methanol with a Y:Ba:Cu ratio of 1:2:3 and a total concentration of 1.08 M L^−1^. Prior to spin-coating, the (100)-oriented recrystallized LaAlO_3_ single crystal substrates were cleaned with isopropanol and heated to 400 °C to improve wettability. The substrates were spin-coated with 2000 rpm for 1 min and subsequently pyrolyzed by heating to 400 °C with a heating rate of 3–5 °C min^−1^ under a humidified O_2_ atmosphere. The pyrolyzed YBCO films were subsequently treated to obtain the desired superconducting film with the high-temperature thermal treatment at 800 °C for 70 min in a humid 200 ppm O_2_ in N_2_ atmosphere which was switched to dry O_2_ at 450 °C for 2 h during the annealing step.

### 2.4. Microstructural Characterization

Texture quality and phase composition of the YBCO thin films were investigated by means of X-ray diffraction (XRD) on a Bruker D4 diffractometer,(Billerica, MA, USA (Cu-K_α_). The distribution of metal ions in amorphous BYF matrix after pyrolysis was determined via Time-of-Flight (ToF) SIMS using a modified TOF-SIMS IV device from ION-TOF GmbH (Münster, Germany), equipped with a 25 kV Bi LMIG and 10 kV C60 sputter source. During the sputtering, the use of C60^++^ clusters with a sputter current of 0.5–2 nA was introduced. The raster size of the sputter beam was set at 300 × 300 µm². Bi^+^-ions were used as primary ions with a pulsed target current between 0.2 and 0.5 pA. The TOF analyzer was set in positive mode.

High-resolution and high annular dark-field scanning transmission electron microscopy (HRTEM and HAADF-STEM) images were taken on a JEOL JEM-2200FS (Tokyo, Japan) TEM with a Cs corrector, operated at 200 kV. For TEM analysis, a cross-sectional TEM lamella was obtained using ion milling techniques via the FIB in-situ lift-out procedure with an Omniprobe™ (FEI, Hillsboro, OR, USA)extraction needle and top cleaning. Chemical information was obtained via the combination of HAADF-STEM with energy dispersive X-ray spectroscopy (EDX).

### 2.5. Electrical Characterization

The self-field critical current density *J*_c_ at 77 K was determined inductively with a 50 µV voltage criterion in a Theva Cryoscan™ system. The magnetic properties were measured with a Quantum Design Physical Property Measurement System (PPMS) with AC-measurement system.( San Diego, CA, USA). The magnetic transition temperature *T*_c_ was defined as the onset temperature of the in-phase component of the AC-magnetization at zero-field in the range of 10–100 K. The width of the magnetic transition was calculated as Δ*T*_c_ = *T*_c,90_ − *T*_c,10_. The DC-measurement was used to determine the critical current of the sample at constant temperature of 77 K as a function of the applied magnetic field perpendicular to the direction of current flow. The *J*_c_’s of all samples are calculated using the Bean critical state model from the opening of the hysteresis loop up to 8 T, obtained by DC-magnetization. The *J*_c_ was recorded with the electric field criterion of 215 µV cm^−1^. The Bean model is widely used because of the ease of use and its accuracy. However, the obtained inductively *J*_c_ values measured via the Cryoscan™ system must be carefully compared with the magnetically *J*_c_ values obtained via PPMS system [28].

## 3. Results and Discussion

**Figure 1:**
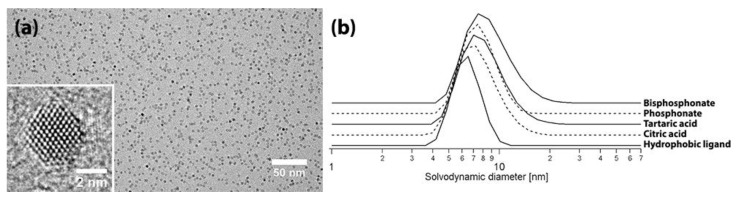
(**a**) Transmission electron microscopy (TEM) image of the ZrO_2_ nanocrystals after the heating-up synthesis (inset shows the structure of the s crystalline grains), (**b**) Dynamic Light scattering (DLS) volume percent analysis of ZrO_2_ nanocrystals before and after ligand exchange with short carboxylate and after ligand exchange with the steric dispersant.

**Figure 2:**
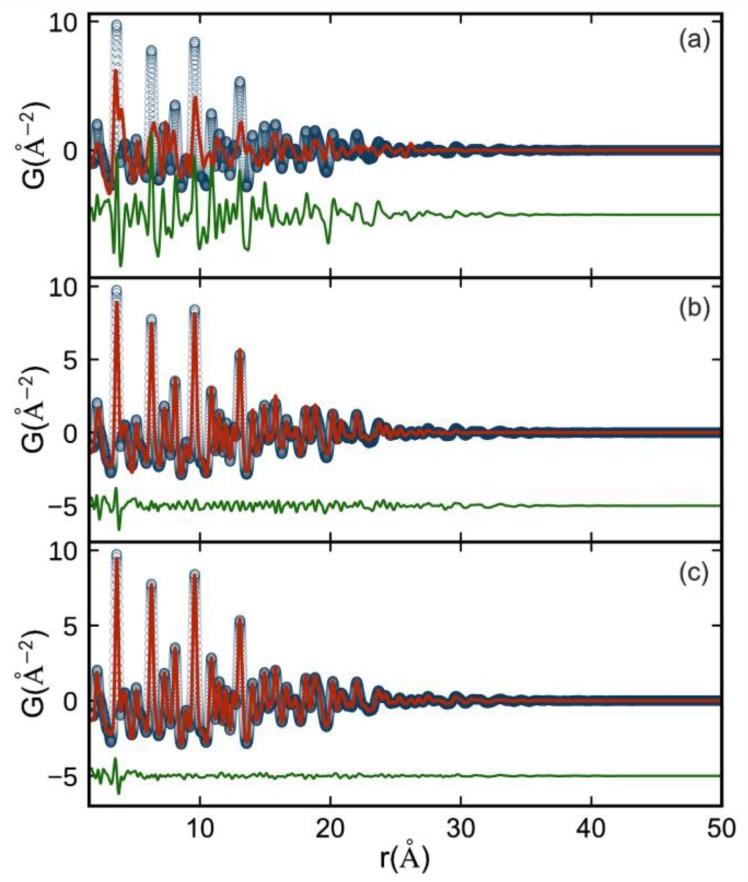
Measured (open circles) and calculated (red solid lines) Pair Distribution Functions (PDFs) with difference curves shown offset below (green) for three candidate ZrO_2_ crystallographic phases fit to an experimental PDF from ∼3.5 nm nanocrystals (**a**) monoclinic (P2_1_/c) (**b**) cubic (Fm-3m) and (**c**) tetragonal (P4_2_/nmc-II). Parameters used for each model are shown in Table 1 and discussed in the text.

We chose to synthesize ZrO_2_ nanocrystals via a heating-up synthesis with tri-*n*-octylphosphine oxide due to their high quality and already known nanocrystal surface chemistry. These nanocrystals are capped with hydrophobic phosphorus-containing ligands including di-*n*-octylphosphinic acid and P,P′-(di-*n*-octyl)pyrophosphonate which are formed upon decomposition of tri-*n*-octylphosphine oxide solvent [20]. The nanocrystals are 3.5 nm in diameter (according to TEM, Figure 1A) and stable in toluene with a solvodynamic diameter of 6.3 nm as measured by DLS (Figure 1B). The monocrystallinity and tetragonal structure of the nanocrystals was supported by atomic pair distribution function (PDF) analysis. In Figure 2 we show single phase refinements of three common ZrO_2_ polymorphs, fitted to an experimental PDF from ∼3.5 nm ZrO_2_ nanocrystals over the full *r*-range where structure can be resolved (1.5 ≤ *r* ≤ 50 Å). The refined parameters per model are provided in Table 1. The monoclinic P2_1_/c phase is ruled out by the agreement factor (R_w_). The cubic-fluorite (Fm-3m) and tetragonal (P4_2_/nmc) structures are similar, but the lower symmetry tetragonal model allows for an independent refinement of the *c*-axis lattice parameter and a displacement of oxygen atoms along the same axis. These models were tested in stages as shown in Table 1. First for P4_2_/nmc-I, where the tetragonality of the unit cell is confirmed by the significant reduction in R_w_ versus the cubic model, and second for P4_2_/nmc-II, where a distortion of the eight-fold coordinated oxygen polyhedra (see Figure S1) further improves the agreement factor, and substantially reduces the average atomic displacement parameters (ADPs) for oxygen atoms. The magnitude of the oxygen displacement from the high symmetry (4*d*) site along the c-axis is ∼0.2 Å. The refined oxygen position for the P4_2_/nmc-II model is robust, and matches the position refined from neutron diffraction studies where oxygen structural parameters can be extracted even more reliably [26]. There is a small structural misfit in the low-*r* region near ∼3.5 Å which may originate from ligand-nanoparticle correlations that are not included in the models. The agreement factor for the P4_2_/nmc-II model is excellent, better than any previously reported agreement factor for a PDF refinement of nanocrystalline ZrO_2_ [29,30,31], and decreases the likelihood of phase coexistence in these samples. Furthermore, given the agreement between the refined PDF crystallite size and supporting particle size estimates, the colloidally stable nanocrystals studied here are fully ordered and tetragonal.

As the YBCO precursor solution is highly ionic and methanol based, a ligand exchange is essential to stabilize the nanocrystals in the precursor solution. According to the previous work, it is possible to stabilize ZrO_2_ nanocrystals in methanol via the addition of short carboxylate (tartaric acid and citric acid) and steric dispersant (a polar copolymer with phosphonate group) [17]. Short carboxylate capped ZrO_2_ nanocrystals show a polydispersity index value of around 0.90 (measured via DLS, Figure 1b, indicating more ZrO_2_ agglomeration) while a copolymer with phosphonate group led to a polydispersity index value of 0.32 [17]. This indicates that phosphonates bind better to the surface than carboxylates, leading to less agglomeration, which is also confirmed in the work of De Keukeleere et al. [20]. As nanocrystals should be agglomerate-free and phosphonates bind better to nanocrystal surfaces, we introduce here a copolymer with bisphosphonate group to stabilize ZrO_2_ nanocrystals even better. The use of this ligand results in a low polydispersity index value of 0.35 and solvodynamic diameter of 8.8 nm as shown in Figure 1b.

We previously characterized the surface chemistry of ZrO_2_ nanocrystals stabilized with citric acid [20] or phosphonate copolymer [17] in methanol. Here, we analyze the binding of the bisphosphonate ligand to the zirconia nanocrystal surface. The ^1^H NMR spectrum of bisphosphonate based ZrO_2_ nanocrystals in methanol-*d*_4_ (Figure S2) shows resonances between 0.7 and 2 ppm attributed to tri-*n*-octylphosphine oxide, di-*n*-octylphosphinic acid, and P,P′-(di-*n*-octyl)pyrophosphonate. The bisphosphonate copolymer resonances are in the range 3.3–4 ppm. In the ^31^P spectrum, sharp resonances are detected for tri-*n*-octylphosphine oxide and di-*n*-octylphosphinic acid while the P,P′-(di-*n*-octyl)pyrophosphonate resonance is too broad to be observed (Figure S2). This indicates that the former two are displaced from the surface by the bisphosphonate while the latter remains bound to the surface. This conclusion is confirmed by the bi-exponential decay of the CH_3_ resonance in Pulsed Field Gradient NMR (Figure S3). The smallest diffusion coefficient of 91 µm² s^−1^ can be converted to a diameter of 8.8 nm. This is somewhat larger than the original solvodynamic size in toluene of 6.3 nm due to the longer chain of the copolymer with bisphosphonate group compared to octyl chains (23 versus 8 atoms). As expected, the diffusion coefficient obtained from the CH_2_ resonances of the copolymer with bisphosphonate group is exactly the same, confirming its strong binding. Thus, the stabilization of ZrO_2_ nanocrystals with ligands containing phosphonate or bisphosphonate groups is more effectively compared to the short carboxylates.

However, the presence of phosphorus in ligands with phosphonate or a bisphosphonate group can lead to the degradation of the YBCO nanocomposite film [17,32] which would degrade the superconducting properties. To study the influence of phosphorus during the YBCO processing, ~1 m% copolymer (containing phosphonate or bisphosphonate) without nanocrystals was added in YBCO precursor solution. The bisphosphonate-containing YBCO has a slightly lower critical current (Table 2, average of 5 samples) compared to the undoped YBCO film while phosphonate-containing YBCO film shows a slight improvement. So, it is clear that the presence of a small amount of phosphorus has no large detrimental effect on the superconducting properties.

**Figure 3:**
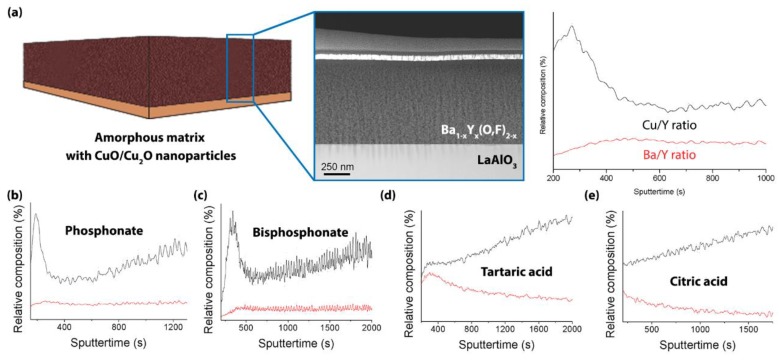
The relative composition of Ba/Y (red line) and Cu/Y (black line) in pyrolyzed amorphous BYF matrix of (**a**) undoped and (**b-e**) ZrO_2_-doped YBCO films, determined via secondary ion mass spectroscopy (SIMS) analysis. Different sputter time steps are due to the introduction of different sputter current.

As the thermal decomposition of the metal organic precursors is one of the critical steps in CSD-based growth, it is important that the stabilizing ligands have only a limited influence on the pyrolysis step [33]. To further unravel the behaviour of the stabilizing ligands, secondary ion mass spectroscopy (SIMS) was introduced to analyze undoped amorphous BYF matrix with CuO nanoparticles after the pyrolysis step (Figure 3A) (black line). This analysis reveals the ratio of Ba/Y (red line) and Cu/Y indicating that more CuO/Cu_2_O nanoparticles are on top of the pyrolyzed matrix with a constant Ba/Y ratio throughout pyrolyzed sample. These large CuO/Cu_2_O particles can lead to undesired phases during the thermal process and thus the degrading *J*_c_ of the resulting films.

**Figure 4:**
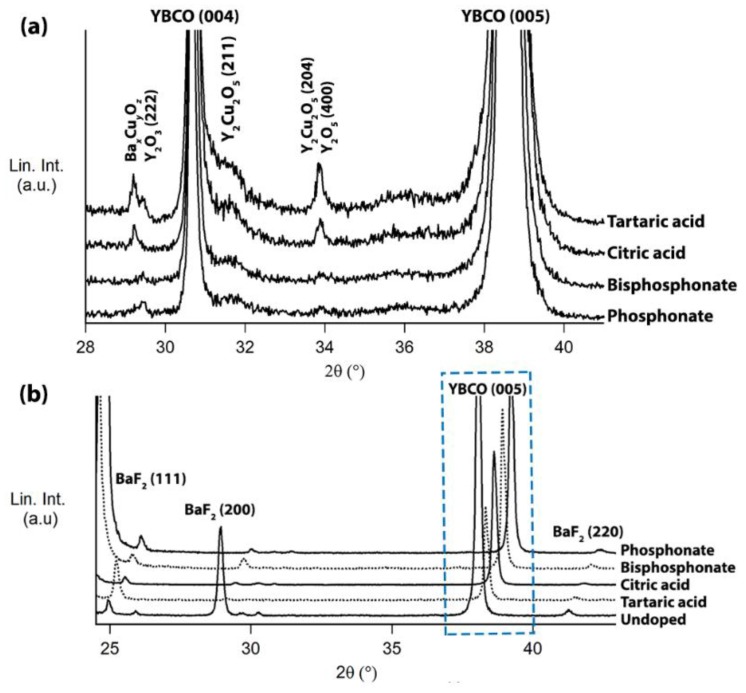
X-ray diffraction (XRD) analysis of (**a**) ZrO_2_-doped YBCO films using different ligands after YBCO crystallization and (**b**) XRD scans of different crystallized YBCO films quenched at 800 °C, indicating the YBCO growth rate (blue rectangle marked) is different. (Reflections marked with an asterisk are related to Ba_x_Cu_y_O_z_ phase.).

We have chosen 5 mol % ZrO_2_-doped pyrolyzed YBCO samples starting from different ligands to study the metal distribution of Y, Ba, and Cu metal ions (Figure 3B–E) in the whole amorphous matrix. SIMS analysis (Figure 3B,C) shows that phosphorus-containing ligands exhibit an amorphous matrix with a constant Ba/Y ratio similar to the undoped matrix. Also on the top-surface, there is a Cu-rich zone for the copolymer with phosphonate and bisphosphonate based ZrO_2_-doped sample. This metal ion distribution in the matrix yields excellent superconducting properties (e.g., high critical current density, *J*_c_). However, short carboxylate based ZrO_2_-doped films show an inhomogeneous distribution of metal ions (i.e., irregular ratio of Ba/Y) into the matrix and result in lower critical currents. It is possible that the quality of YBCO structure is sensitive to the inhomogeneity of metal content (especially Ba and Y) in the layer. To unravel this effect, all pyrolyzed samples underwent a YBCO crystallization process and were analyzed via x-ray diffraction (XRD, Figure 4A). Based on XRD patterns, short carboxylate based nanocomposite films contain more secondary phases such as Ba_x_Cu_y_O_z_ at 2θ = 29.3° and Y_2_Cu_2_O_5_—(211) at 2θ = 31.5° and (204) at 2θ = 33.4°—while steric dispersant based films have minor secondary phases. This may be due to the disturbed nucleation mechanism of the epitaxial YBCO film because the formation of the BaF_2_ phase on the LaAlO_3_ interface is not beneficial. Also SIMS indicates there are fewer Ba^2+^ present at the bottom layer of the pyrolyzed layer. This is also confirmed via YBCO (005) reflections (2θ = 38.5, Figure 4B) of crystallized samples quenched at 800 °C, indicating that the nucleation/growth mechanism is different. The XRD spectrum (Figure 3B) features crystalline BaF_2_ that is in the process of reacting towards epitaxial YBCO. The difference in BaF_2_ intensities is due to the competing reaction with ZrO_2_ nanocrystals in combination with less availability of Ba^2+^ at LaAlO_3_ interface during the nucleation process.

**Figure 5:**
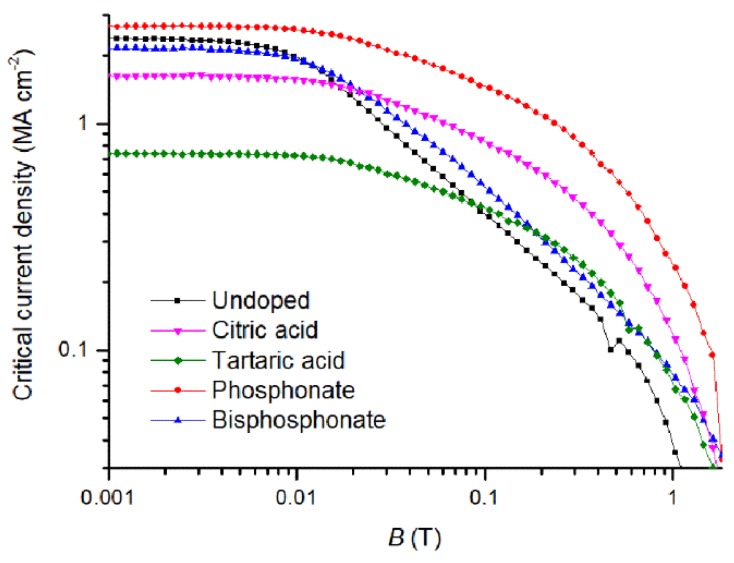
Double logarithmic plots of critical current density vs. magnetic field *H* measured at 77 K for undoped and ZrO_2_-doped YBCO films on LaAlO_3_ substrates.

**Figure 6:**
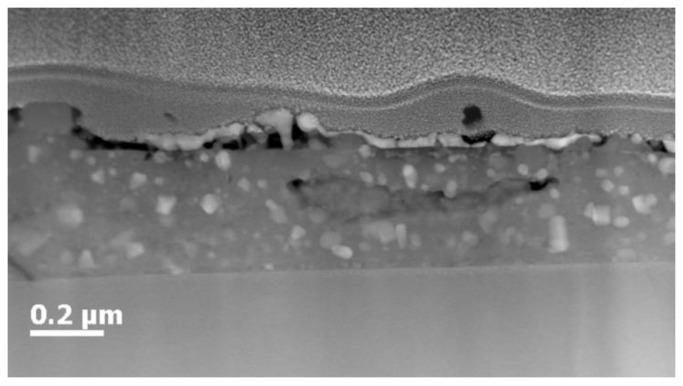
High annular dark-field scanning transmission electron microscopy (HAADF-STEM) cross sectional image of tartaric acid based ZrO_2_-doped YBCO film, indicating lots of secondary phases in YBCO matrix.

Good biaxial YBCO texture is important but it is essential that the nanocrystals are incorporated into YBCO matrix to deliver good pinning properties. For this reason, the magnetic properties were measured with a PPMS. The onset magnetic transition (*T*_c_) values and *J*_c_ are listed in Table 3. These magnetically measured transitions are very informative to understand the overall film quality due to the current percolation throughout the YBCO film. It can be concluded that the addition of nanocrystals hardly influence the magnetic transition. However, the tartaric acid based ZrO_2_-doped YBCO film shows a slight decrease with wider width of *T*_c_ and is probably explained by a multitude of the secondary phases in YBCO matrix as shown in XRD analysis (Figure 4A). On the other hand, the magnetic field dependences of *J*_c_’s calculated at 77 K in maximum Lorentz force configuration are shown in Figure 5. It is clear that the critical current densities are in the range of 1.5–3 MA cm^−2^ except tartaric acid based ZrO_2_-doped YBCO film, which only achieved a *J*_c_ of 0.74 MA cm^−2^. The latter is due to a large amount of undesired secondary phases in the YBCO matrix as confirmed on HAADF-STEM image (Figure 6) and XRD analysis (Figure 4A).

To determine if the ZrO_2_ nanocrystals in YBCO matrix act as pinning centers, we studied the shape of the *J*_c_(H) curves in Figure 5. Copolymer with phosphonate, citric acid, and tartaric acid based ZrO_2_-doped YBCO films (except copolymer with bisphosphonate) show a smoother decay—round shape of *J*_c_(*B*)—compared to undoped YBCO film. This is also confirmed by higher values of accommodation field B* (determined by the criterion $J_{c}\left(B^{*}\right)=0.9J_{c}\left(0\right)$ at 77 K) and by lower values of the slope (power-law exponent α) in log–log plot (Table 3). The low field plateau below B* is the single vortex pinning region where each vortex is pinned to a free pinning site [34]. At B* collective pinning effects take place and a reduction of the slope of α is seen in log–log plot. (Figure 5) The α values were estimated and are lower than 0.5, indicating that the pinning site (sizes comparable to the coherence length) is strong enough to destroy the vortex lattice and pin the vortices individually [34]. However, bisphosphonate based ZrO_2_-doped YBCO film shows a straight shape of *J*_c_(*B*) and thus also results in a higher α value of 0.5. It is explained due to the presence of very large BaZrO_3_ particles in the YBCO matrix (vide infra).

The values of B* and α are listed in Table 2. A clear difference between undoped and ZrO_2_-doped films (except for the copolymer with bisphosphonate group) can be seen. This means that the addition of preformed nanocrystals increases pinning (leading to higher B* values) and results in a slower decay (leading to lower α values) of critical current density in the function of the magnetic field at 77 K. However, the *J*_c_ value of tartaric acid-based ZrO_2_-doped YBCO nanocomposite is still lower than any other ZrO_2_-doped films and is accompanied by the presence of more undesired secondary phases in the YBCO matrix as confirmed via XRD (Figure 3A) and the HAADF-STEM image (Figure 5).

**Figure 7:**
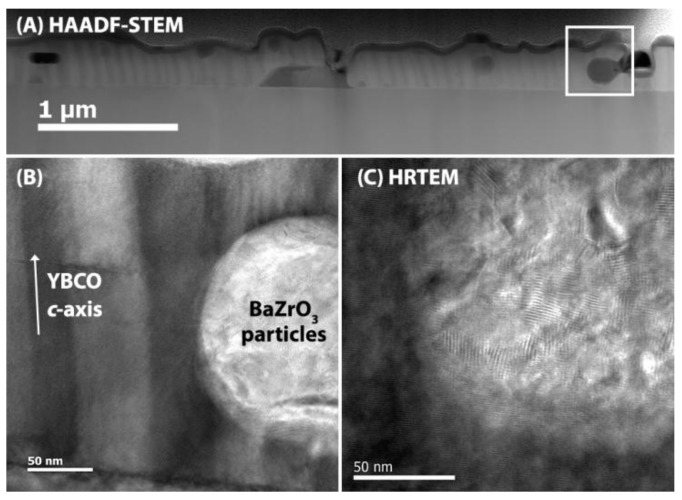
(**a**) HAADF-STEM image of copolymer with bisphosphonate capped ZrO_2_-doped YBCO film grown on LaAlO_3_ substrate, indicating big particles in the YBCO matrix, (**b**) TEM image showing YBCO/BaZrO_3_ particles interface and (**c**) High-resolution transmission electron microscopy (HRTEM) image of BaZrO_3_ particles, showing a coagulation of ZrO_2_ nanocrystals.

It is remarkable that the bisphosphonate based ZrO_2_-doped YBCO nanocomposite did not show any improvement of pinning behaviour while the self-field *J*_c_ has an acceptable value in the range of 2–2.5 MA cm^−2^. As shown on the HAADF-STEM image (Figure 7A) and TEM image (Figure 7B), the YBCO layer is strongly textured and grows epitaxially on the LaAlO_3_ substrates which explains the good superconducting properties. However, some secondary phases and large BaZrO_3_ particles in the size of 150–200 nm can be observed in the YBCO layer via EDX analysis. In order to have a better understanding of the BaZrO_3_ particles in the YBCO matrix, HRTEM analysis was used. Figure 7C shows that this BaZrO_3_ particle contains several aggregated small nanocrystals. So, it seems that the nanocrystals are coagulated during the YBCO growth to the size of approximately 200 nm. This particle is too large to act as a pinning center because it is not in the order of superconducting coherence length of 2–4 nm for YBCO at 77 K. It appears that the stabilizing effect of bisphosphonate copolymer is reduced or annihilated upon the initial decomposition, leading to an increased tendency to form agglomerations. However, the precise origins of this effect remain unclear.

## 4. Conclusions

ZrO_2_ nanocrystals, synthesized in tri-n-octylphosphine oxide were probed with PDF analysis and found to be monocrystalline, featuring a distorted tetragonal crystal structure. They were subsequently stabilized by a steric polar ligand (Copolymer with phosphonate or bisphosphonate group) or short carboxylates (tartaric and citric acid) in low-fluorine YBCO precursor solutions. From these suspensions, we synthesized high quality superconducting ZrO_2_-doped YBCO nanocomposites in a single coating step, in contrast to earlier results with amino acid stabilized nanocrystals. Interestingly, nanocrystals stabilized by short carboxylate ligands resulted in poorly superconducting nanocomposites while the phosphonate dispersant led to excellent self-field superconducting nanocomposites. This is a counter-intuitive result as one would expect that as phosphorus or/and carbon is introduced in the layer, the worse the superconductor would be. The use of copolymer with bisphosphonate group results in the coagulation of ZrO_2_ nanocrystals during YBCO growth, resulting in no improved pinning properties. Given the counter-intuitive relation between the nanocrystal surface chemistry and the final nanocomposites performance, we expect that surveying a wide library of ligands will be crucial in order to obtain a good superconducting nanocomposite film with the ability to pin the vortices. The use of a copolymer with bisphosphonate group leads to coagulation of ZrO_2_ nanocrystals during YBCO growth likely due to the reduction of stabilizing effects during the thermal decomposition, which do not facilitate pinning.

## Figures and Tables

**Table 1:** Refined parameters from virtual crystal nanoparticle ZrO_2_ models. P4_2_/nmc-II differs from P4_2_/nmc-I due to an additional degree of freedom which allows oxygen atom displacement off of the 4*d* Wyckoff position as described in the text. The crystallite size is refined from a spherical shape function parameter and refers to the domain of coherent scattering in the material.

	P2_1_/c	Fm-3m	P4_2_/nmc-I	P4_2_/nmc-II
a (Å)	5.200	5.125	3.603	3.603
b (Å)	5.231	5.125	3.603	3.603
c (Å)	5.617	5.125	5.188	5.186
β	94.8	90.0	90.0	90.0
Zr-Uiso (Å)	0.008	0.010	0.008	0.009
O-Uiso (Å)	0.046	0.072	0.072	0.041
Crystallite size (Å)	36.9	34.2	38.8	39.1
z(O1) (f.c.)	–	0.25	0.50	0.45
R_w_	0.737	0.151	0.120	0.098

**Table 2:** Thickness and its critical current of undoped YBa_2_Cu_3_O_7−δ_ (YBCO) film without and with 1 m% phosphonate-containing copolymer.

Ligands	Thickness (nm)	Critical Current, *I*_c_ (A)
Undoped	275 ± 14	139 ± 25
Phosphonate	280 ± 10	144 ± 17
Bisphosphonate	282 ± 17	129 ± 14

**Table 3:** Collection of magnetic transition temperature *T*_c_ and its width, critical current densities *J*_c_ at self-field and 1 T, accommodation field B* and the power-law exponent α in undoped and ZrO_2_-doped YBCO films on LaAlO_3_ substrates.

Ligands	*T*_c_ (K)	Δ*T_c_* (K)	*J*_c,mag_ (0 T) (MA cm^−2^)	*J*_c,mag_ (1 T) (kA cm^−2^)	B* (mT)	α
Undoped	90.0	1.1	2.37	41.54	7.62	0.68
Phosphonate	90.5	1.5	2.68	237.00	17.02	0.39
Bisphosphonate	91.5	1.6	2.14	79.05	9.85	0.58
Citric acid	90.5	2.2	1.65	120.32	15.52	0.40
Tartaric acid	89.0	2.5	0.74	72.76	20.06	0.40

## Supplementary Materials

The following are available online at http://www.mdpi.com/1996-1944/11/7/1066/s1, Figure S1: tetragonal and distorted tetragonal crystal structure for ZrO_2_ after PDF refinement. Figure S2: ^1^D ^1^H spectrum and (B) ^31^P spectrum of ZrO_2_ nanocrystals stabilized with bisphosphonate (BP) in MeOD-*d*_4_, with (C) a zoom on BP resonances. Figure S3: Bi-exponential diffusion decay fitting of the bisphosphonate ligand.
