# Supplementary material for: Pair Distribution Function Analysis of ZrO2 Nanocrystals and Insights in the Formation of ZrO2-YBa2Cu3O7 Nanocomposites
Source: Materials (Basel). 2018 Jun 23;11(7):1066. doi: 10.3390/ma11071066 (PMC6073135; doi:10.3390/ma11071066)
Supplement: Supplementary file 1 [file materials-11-01066-s001.pdf]

Supplementary Materials: Pair Distribution Function Analysis of ZrO_2 Nanocrystals and Insights in the Formation of ZrO_2-$YBa_2Cu_3O_7$ Nanocomposites

Hannes Rijckaert [1], Jonathan De Roo [1], Matthias Van Zele [1], Soham Banerjee [2], Hannu Huhtinen [3], Petriina Paturi [3], Jan Bennewitz [4], Simon J. L. Billinge [2,5], Michael Bäcker [6], Klaartje De Buysser [1] and Isabel Van Driessche [1,*]

1 Ghent University, SCRiPTS, Dept. of Chemistry, Krijgslaan 281-S3, 9000 Ghent, Belgium; Hannes.Rijckaert@ugent.be (H.R.); Matthias.VanZele@ugent.be (M.V.Z.); Jonathan.DeRoo@ugent.be (J.D.R.); Klaartje.DeBuysser@ugent.be (K.D.B.)
2 University of Turku, Wihuri Physical Laboratory, Dept. of Physics and Astronomy, 20014 Turku, Finland; Hannu.Huhtinen@utu.fi (H.H.); Petriina.Paturi@utu.fi (P.P.)
3 BASF SE, Advanced Materials & Systems Research, Carl-Bosch-Straße 38, 67056, Ludwigshafen am Rhein, Germany; Jan.Bennewitz@basf.com (J.B.)
4 Dept of Applied Physics and Applied Mathematics, Columbia University, 1105 S.W. Mudd, New York, NY 10027; sb3519@columbia.edu (S.B.); sb2896@columbia.edu (S.B.)
5 Deutsche Nanoschicht GmbH, Heisenbergstraβe 16, 53359 Rheinbach, Germany; Baecker@d-nano.com (M.B.)
* Correspondence: Isabel.VanDriessche@ugent.be; Tel.: +32-9263-4433

Refined crystal structure

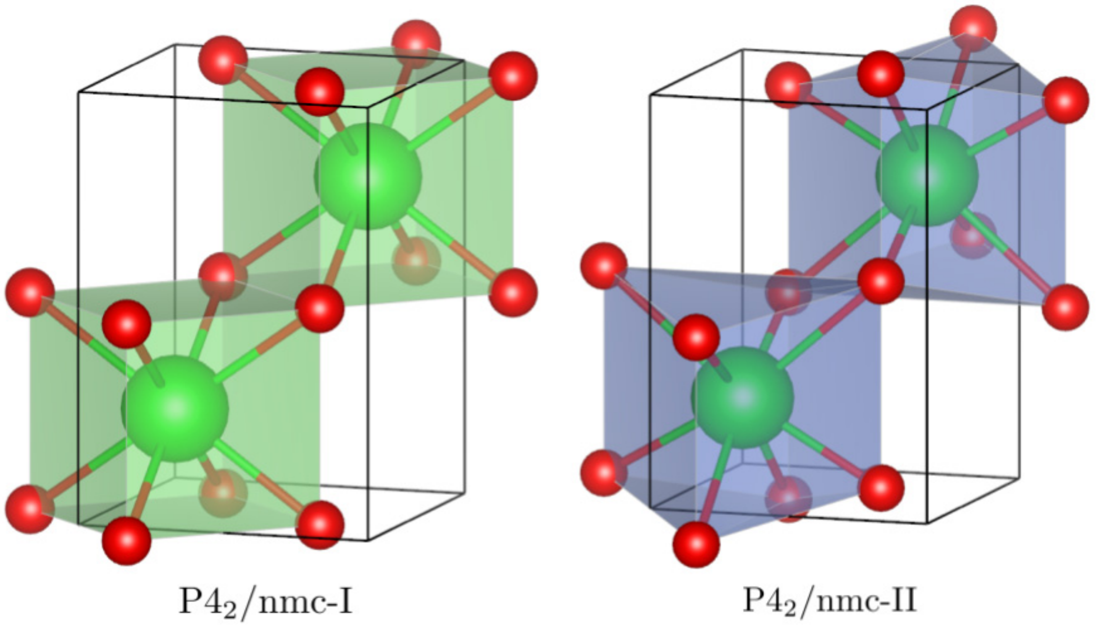

Figure S1. (left) tetragonal and (right) distorted tetragonal crystal structure for ZrO_2 after PDF refinement.

Nuclear Magnetic Resonance analysis of the bisphosphonate stabilized nanocrystals

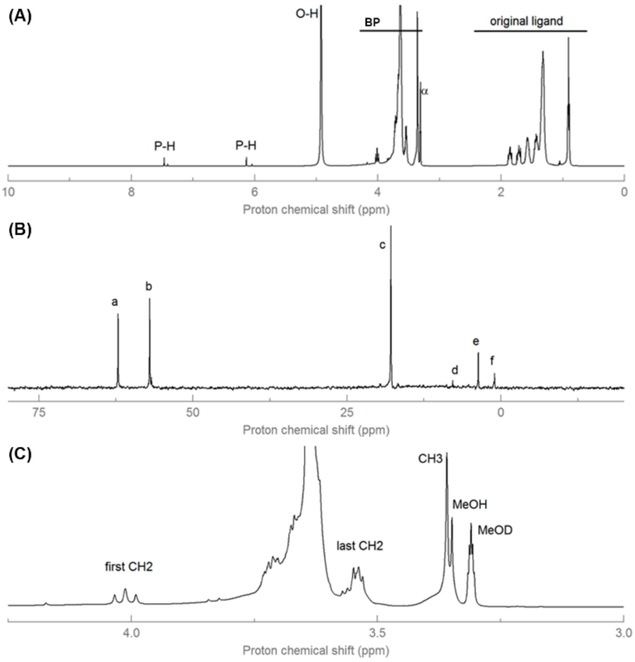

Figure S2. (A) 1D ^{1}H spectrum and (B) ^{31}P spectrum of ZrO_2 nanocrystals stabilized with bisphosphonate (BP) in MeOD-d_4, with (C) a zoom on BP resonances.

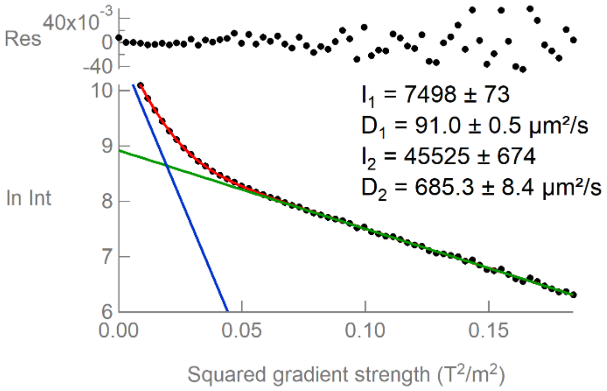

Figure S3. Bi-exponential diffusion decay fitting of the bisphosphonate ligand.
